# Improvement of the outcome of the saphenous vein graft when connected to the internal thoracic artery

**DOI:** 10.3389/fcvm.2024.1478166

**Published:** 2024-10-18

**Authors:** Konstantinos Katsavrias, Sotirios Prapas, Antonio M. Calafiore, David Taggart, Dimitrios Angouras, Dimitrios Iliopoulos, Michele Di Mauro, Styliani Papandreopoulos, Panayiotis Zografos, Dimitrios Dougenis

**Affiliations:** ^1^ 1st Department of Cardiac Surgery, Henry Dunant Hospital, Athens, Greece; ^2^ Department of Cardiac Surgery, John Radcliffe Hospital, University of Oxford, Oxford, United Kingdom; ^3^ Department of Cardiothoracic Surgery, Medical School of the National and Kapodistrian University, Athens, Greece; ^4^ Cardio-Thoracic Surgery Unit, Heart and Vascular Centre, Maastricht University Medical Centre (MUMC), Cardiovascular Research Institute Maastricht (CARIM), Maastricht, Netherlands; ^5^ Department of Cardiology, Pierangeli Hospital, Pescara, Italy

**Keywords:** I-graft, composite graft, saphenous vein graft, anaortic bypass, OPCAB

## Abstract

**Background:**

Since 2000, we have been grafting the right coronary artery system (RCAs) using the proximal portion of the right internal thoracic artery (RITA) as the inflow of the saphenous vein graft (SVG) to increase the number of patients undergoing beating heart complete myocardial revascularization.

**Methods:**

From 2000 to 2022, 928 consecutive patients underwent SVG on the RCAs. In 546 patients (58.8%), the inflow was the RITA (I-graft group), and in 382 patients (41.2%), the inflow was the aorta (Ao-graft group). The inclusion criteria were age ≤75 years, ejection fraction >35%, only one SVG per patient, bilateral internal thoracic arteries as a Y-graft on the left system (three-vessel disease, *n* = 817, 88.0%) or left internal thoracic artery on the left anterior descending artery and RITA + SVG on the RCAs (two-vessel disease, *n* = 111, 12.0%). Propensity matching identified 306 patients per group. After a median follow-up of 8 (5–10) years, graft patency was assessed by coronary computed tomographic angiography in 132 patients (64 in the I-graft group and 68 in the Ao-graft group).

**Results:**

Early results were similar in both groups. The I-graft group had higher 10-year survival and freedom from main adverse cardiac events (90.0 ± 2.0 vs. 80.6 ± 3.8, *p* = 0.0162, and 81.3 ± 2.7 vs. 64.7 ± 5.6, *p* = 0.0206, respectively). When RITA was the inflow, SVG had a higher estimated 10-year patency rate (82.8% ± 6.5 vs. 58.8% ± 7.4, *p* = 0.0026) and a smaller inner lumen diameter (2.7 ± 0.4 vs. 3.4 ± 0.6 mm, *p* < 0.0001).

**Conclusion:**

When the inflow is the RITA, SVG grafted to the RCAs (I-graft) may result in a higher patency rate and better outcome than when the inflow is the ascending aorta (Ao-graft). The continuous supply of nitric oxide by RITA may be the cause of the higher patency rate of the I-graft, which can behave like an arterial conduit.

## Introduction

Although the results of surgical myocardial revascularization using multiple arterial conduits (ACs) seem to be better than the use of a single left internal thoracic artery (LITA) supplemented by saphenous vein grafts (SVGs), the prevalence of the former strategy is still far from widespread acceptance today. The superiority of multiple ACs is mainly based on propensity matching studies, which, however, have intrinsic biases and unmeasured confounding factors that are not eliminated by the statistical method (1). In addition, the most recent ART trial failed to demonstrate a benefit in terms of 10-year survival or freedom from death, myocardial infarction, and stroke (2) in patients with bilateral internal thoracic arteries (BITA) or LITA with SVGs or radial artery (RA). Although a new randomized controlled trial, the ROMA trial (3), is currently underway to address some of the drawbacks of the ART trial, such as the high percentage of crossover from the BITA to the single internal thoracic artery and the possibility of using the RA, the SVG remains the most frequently used conduit during surgical myocardial revascularization.

The 1-year failure rate of SVG has decreased in recent decades and is now 10%–12%. These results have been achieved because it has been recognized that improving harvesting techniques, reducing dilation pressure, and selecting more appropriate storage solutions are critical to minimizing SVG damages. Various surgical strategies have also been used to improve the patency rate of SVG, such as the no-touch technique (4, 5), the use of external stents (6), and proximal anastomosis to the LITA as a Y-graft (7). To extend anaortic myocardial revascularization to a larger number of patients, we have been using SVG anastomosed end to end to the stump of the right internal thoracic artery (RITA) to graft the right coronary artery system (RCAs) for more than 20 years (8, 9). We then decided to compare this strategy with the conventional use of a SVG anastomosed proximally to the ascending aorta when the RCAs needs to be revascularized, to find out whether the clinical outcomes and patency rate of the SVG anastomosed to the RITA are better than when the SVG has the ascending aorta as its blood source.

## Materials and methods

Coronary artery bypass grafting (CABG) on the beating heart by median sternotomy was performed on 5,560 patients at the Henry Dunant Hospital in Athens from January 2020 to December 2022. Patients were included in this study based on the following criteria: age ≤75 years, ejection fraction >35%, need to graft the RCAs in the setting of two- or three-vessel disease, use of BITA, and use of only one SVG to graft the RCAs without additional SVG to the anterior or lateral wall. By excluding patients older than 75 years and patients with an ejection fraction of ≤35%, we were able to better assess the impact of the procedure on long-term outcomes and avoid two important risk factors for reduced survival. The introduction of the I-graft (RITA + SVG) was due to the effort to expand anaortic myocardial revascularization, one of the team's goals. The introduction was gradual, as the ascending aorta was still used as a blood source in many patients, especially in the early years, depending on the surgeon's preference. The Institutional Review Board approved this retrospective observational study on 8 October 2020 (n.68) and waived patient consent.

### Surgical technique

In all patients, myocardial revascularization was performed on a beating heart via a median sternotomy. The ITAs were harvested skeletonized. The LITA was always left *in situ*. In patients with three-vessel disease, the RITA was cut and used as a Y-graft from the LITA, but its proximal part (∼5 cm). This was used as a blood source for the SVG, which was anastomosed end to end and then clipped distally. In patients with two-vessel disease involving the left anterior descending artery (LAD), and the RCAs, the RITA was harvested at a length of ∼10 cm and then anastomosed end to end to the SVG. In both cases, the vein was dilated by physiologic RITA flow.

The SVG was harvested from the lower leg in the least traumatic way possible, washed with saline, and stored in heparinized blood with papaverine. Endoscopic harvesting was never used. Mechanical distension was as gentle as possible when the SVG was anastomosed proximally to the ascending aorta. When the inflow was the RITA, any mechanical distension was avoided.

### Follow-up

All patients underwent clinical follow-up in our outpatient clinic at 3, 6, and 12 months after surgery and at annual intervals thereafter. The most recent information was obtained by calling the patients or the referring cardiologists. Follow-up ended in September 2023 and was 89% complete. When it was not possible to contact the patients (102 patients, 11.0%), the most recent information was included in the analysis. The median follow-up time of survivors was 7.3 (2.6–12.5) years.

The patency rate was assessed by coronary computed tomographic angiography (CCTA) in 132 patients with unclear symptoms or elective after a median follow-up of 93 (67–98) months. We asked all patients with normal renal function to undergo CCTA to assess graft patency. Most of them refused, either due to economic reasons or because of their age or because they lived outside Athens.

Graft patency was graded as described by FitzGibbon et al. (10). Grade A (excellent) and Grade B (intermediate) grafts were treated as open. Grade O included grafts with stenosis of 75% or more of vessel diameter or complete occlusion and were considered occluded.

### End points

The primary endpoint was 10-year survival and freedom from the main adverse cardiac event, MACE (cardiovascular death, non-fatal myocardial infarction, and need for further myocardial revascularization, either surgical or percutaneous).

### Statistical analysis

The results are expressed as mean and standard deviation or median with 25th and 75th percentiles. The categorical variables are described as number and percentage. Student's *t*-test or Mann–Whitney *U* test (continuous variables) and a *χ*^2^ test or Fisher's exact test (categorical variables) were used to assess the differences between the two groups. Two groups of propensity-matched patients were identified after building a logistic regression model using I-graft as the target variable. A preliminary evaluation of common support was performed using histograms and box plots of the distributions of linear propensity scores for the treated and untreated. Subsequently, a greedy nearest-neighbor with a caliper distance of 0.2 was chosen. The matching was validated by calculating the standardized mean difference, expressed as a percentage. A mean difference of 10% or less was taken as the threshold for a good balance. The risk factors for early mortality were analyzed by stepwise logistic regression analysis. Time-depending events, patency rate included, were evaluated by the Kaplan–Meier method and included the early events. The two groups were compared using log-rank in Kaplan–Meier curves. The variables were defined as in the EuroSCORE II model. The variables included in the model and in the propensity matching are listed in Table 1.

**Table 1:** Preoperative and perioperative data.

	All patients			Propensity-matched		
	I-graft group	Ao-graft group	SMD (%)	I-graft group	Ao-graft group	SMD (%)
*N*	546	382		306	306	
Age	70 ± 10	62 ± 8	37.1	65 ± 8	66 ± 4	−5.7
Female gender	72 (13.2)	48 (12.6)	1.8	51 (16.8)	48 (15.6)	3.2
Emergency	42 (7.7)	19 (5.0)	11.1	18 (5.9)	13 (4.2)	7.8
Unstable angina	69 (12.6)	31 (8.1)	14.8	36 (11.8)	27 (8.8)	9.9
Ejection fraction						
>50%	352 (64.4)	269 (70.4)	−13.0	207 (67.6)	210 (68.6)	−2.1
36%–50%	194 (35.6)	113 (29.6)	13.0	99 (32.4)	96 (31.4)	2.1
Diabetes	140 (25.6)	124 (32.5)	−13.9	84 (27.5)	93 (30.4)	−6.4
Obesity (BMI ≥ 30)	177 (32.4)	107 (28.0)	9.4	79 (25.8)	71 (23.2)	6.0
Smokers	354 (64.7)	228 (59.7)	10.3	168 (54.9)	159 (52.0)	5.8
PVD	59 (10.8)	26 (6.8)	13.8	26 (8.5)	19 (6.2)	8.8
Hyercholesterolemia	285 (52.1)	237 (62.0)	−20.1	176 (57.2)	185 (60.0)	−5.7
Hypertension	302 (55.2)	202 (52.9)	4.6	169 (55.2)	161 (52.6)	5.2
Creatinine	1.01 ± 0.47	0.99 ± 0.43	0.1	1.03 ± 0.51	1.00 ± 0.43	0.2
Creatinine clearance (ml/min)	83 ± 36	88 ± 29	−23.2	83 ± 28	84 ± 26	−5.7
Renal impairment						
No (>85 ml/min)	244 (44.7)	210 (55.0)	−20.9	134 (43.8)	143 (46.7)	−5.8
Moderate (50–85 ml/min)	254 (46.5)	146 (38.2)	16.6	145 (47.4)	142 (46.4)	−2.0
Severe (<50 ml/min)	48 (8.8)	26 (6.8)	8.5	27 (8.8)	21 (6.8)	7.5
On dialysis	2 (0.4)	3 (0.9)	−7.8	1 (0.4)	2 (0.8)	−5.2
Preop AF	5 (0.9)	6 (1.6)	−6.3	4 (1.3)	5 (1.6)	2.5
Preop stroke	11 (2.0)	6 (1.6)	3.0	7 (2.3)	6 (2.0)	2.1
Preop AMI	213 (38.9)	173 (45.3)	−13.0	125 (40.8)	133 (43.5)	−5.6
COPD	46 (8.4)	17 (3.2)	22.4	27 (6.4)	16 (4.0)	10.8
Preop IABP	7 (1.3)	3 (0.8)	4.9	4 (1.3)	3 (1.0)	2.8
Redo	21 (3.8)	9 (2.4)	−8.1	15 (4.9)	8 (2.6)	12.1
LM disease	106 (19.4)	56 (14.7)	12.5	58 (18.9)	45 (14.7)	11.2
Two-vessel disease	70 (12.8)	41 (10.7)	6.5	40 (13.1)	36 (11.8)	3.9
Three-vessel disease	476 (87.2)	341 (89.3)	−6.5	266 (86.9)	270 (88.2)	−3.9
Distal anastomoses	3.2 ± 0.9	3.3 ± 0.8	−0.5	3.4 ± 0.6	3.4 ± 0.6	0
Arterial anastomoses	2.3 ± 0.8	2.4 ± 0.8	−0.5	2.4 ± 0.6	2.4 ± 0.6	0
Conversions	2 (0.4)	2 (0.5)	−1.5	1 (0.4)	1 (0.4)	0

SMD, standardized mean difference; BMI, body mass index; PVD, peripheral vascular disease; preop, preoperative; AF, atrial fibrillation; AMI, acute myocardial infarction; COPD, chronic obstructive pulmonary disease; IABP, intra-aortic balloon pumping; LM, left main.

For all tests, a *p*-value of <0.05 was considered significant. RStudio version 1.1.463 (2009–2018) was used for all statistical evaluations.

## Results

Nine-hundred and twenty-eight consecutive patients met the inclusion criteria. The blood source was the RITA (I-graft group) in 546 patients (58.8%) and the ascending aorta (Ao-graft group) in the remaining 382 patients (41.2%). The preoperative and perioperative data of all patients are listed in Table 1. After propensity matching, 612 patients were included in this study (306 per group) (Table 1).

### Early results

Three-vessel disease was present in 476 patients (87.2%) and two-vessel disease (always LAD and RCAs) in 70 patients (12.8%). Surgery was performed in all cases off-pump. Conversion to cardiopulmonary bypass and cardioplegic arrest occurred in four patients (0.4%), each time electively, because of hemodynamic instability. Early postoperative complications, which were similar in both groups, are shown in Table 2. Thirty-day mortality was low (2/712, 0.3%) and similar in both groups (Table 2). The patients in the Ao-graft group had a higher, but not statistically significant, stroke rate (1.0% vs. 0.3%). The prevalence of atrial fibrillation and length of stay were higher in the I-graft group, without being statistically significant. Multivariable logistic regression did not reveal any risk factor for early mortality due to the low number of events.

**Table 2:** Postoperative complications in propensity-matched patients.

	I-graft group	Ao-graft group	*p*
*N*	306	306	
Stroke	1 (0.3)	3 (1.0)	0.3161
AMI	2 (0.6)	1 (0.3)	0.5631
New IABP	1 (0.3)	2 (0.6)	0.5631
Early AKI	8 (2.6)	8 (2.6)	1.0000
CRRT	0	1 (0.3)	0.3173
Pneumonia	11 (3.6)	6 (2.0)	0.2191
Ventilation >48 h	8 (2.6)	8 (2.6)	1.0000
Atrial fibrillation	51 (16.7)	42 (13.7)	0.3113
Redo for bleeding	7 (2.3)	8 (2.6)	0.7939
Sternal wound complications	5 (1.6)	8 (2.6)	0.4007
Thirty-day mortality	1 (0.3)	1 (0.3)	1.0000
LOS (days)	7.0 ± 2.6	6.6 ± 2.5	0.0529

AMI, acute myocardial infarction; IABP, intra-aortic balloon pumping; AKI, acute renal insufficiency; CRRT, continuous renal replacement therapy; LOS, length of stay.

### Late results

After a median of 8.7 years (3.0–13.9), 89 patients (14.6%) died, of whom 38 (42.7%) died from cardiac causes [acute myocardial infarction (AMI) and its complications]. The non-cardiac causes of death occurred in 51 patients (57.3%): malignancy in 25, cerebral degenerative disease in 8, cerebrovascular accident in 7, chronic obstructive pulmonary disease in 5, abdominal aneurysm rupture in 2, sepsis in 2, car accident in 1, and unknown in 1.

**Figure 1:**
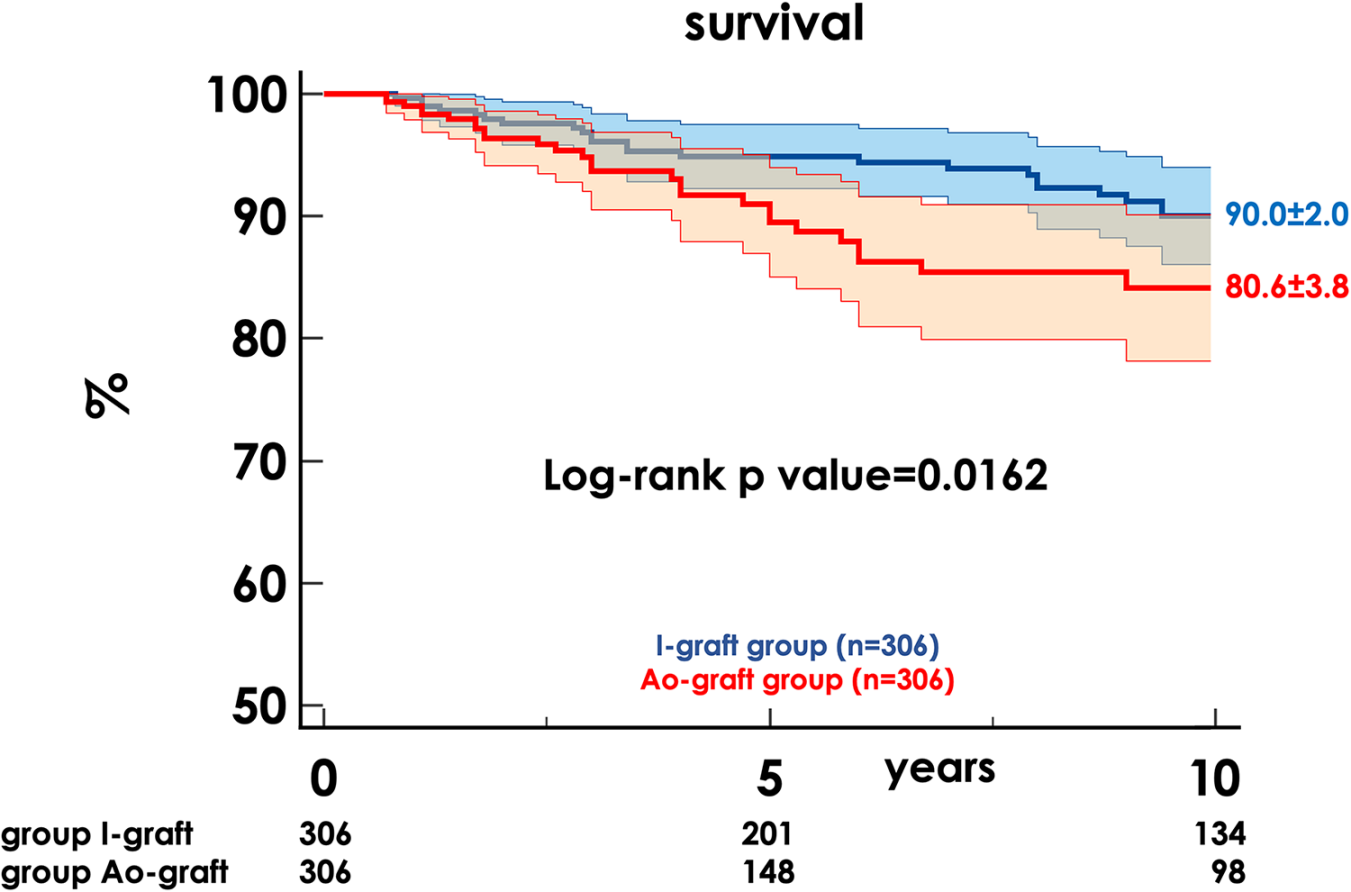
Freedom from death of any cause in the two propensity-matched groups. HR, hazard ratio; CI, confidence interval.

The 10-year survival rate was 86.6 ± 1.6 and was higher in the I-graft group (90.0% ± 2.0 vs. 80.6% ± 3.8, *p* = 0.0162) (Figure 1).

**Figure 2:**
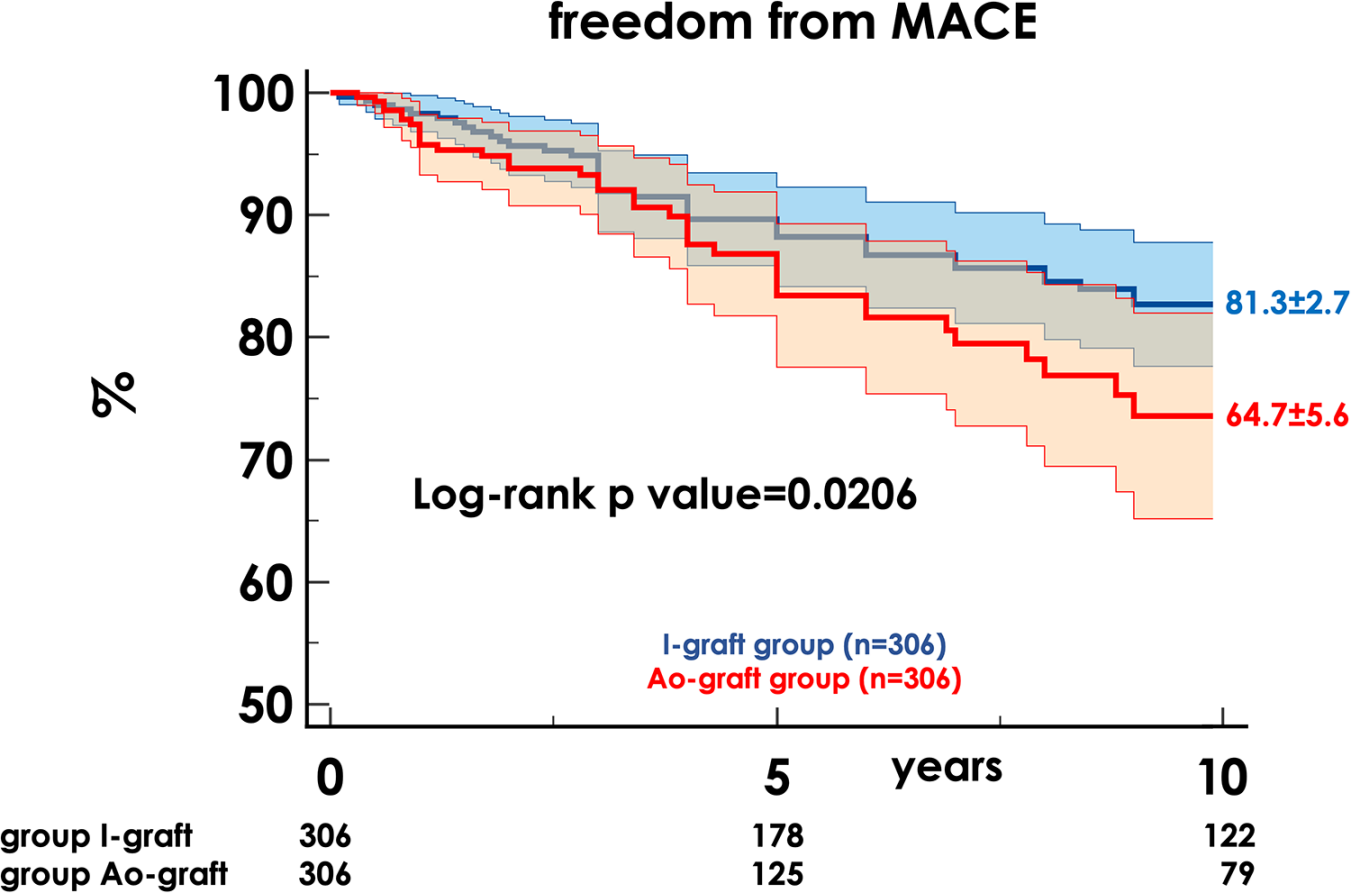
Freedom from MACE in the two propensity-matched groups. MACE, main adverse cardiac events; HR, hazard ratio; CI, confidence interval.

After a median of 4.0 years (1.8–8.2), MACE occurred in 95 patients (15.7%): 39 cardiovascular deaths, 22 non-fatal AMI and 35 myocardial revascularizations, 34 percutaneous, and 1 surgical. The 10-year freedom from MACE was 76.4% ± 2.5 and was higher in the I-graft group (81.3% ± 2.7 vs. 64.7% ± 5.6, *p* = 0.0206) (Figure 2).

### Late evaluation of I-grafts and Ao-grafts

**Figure 3:**
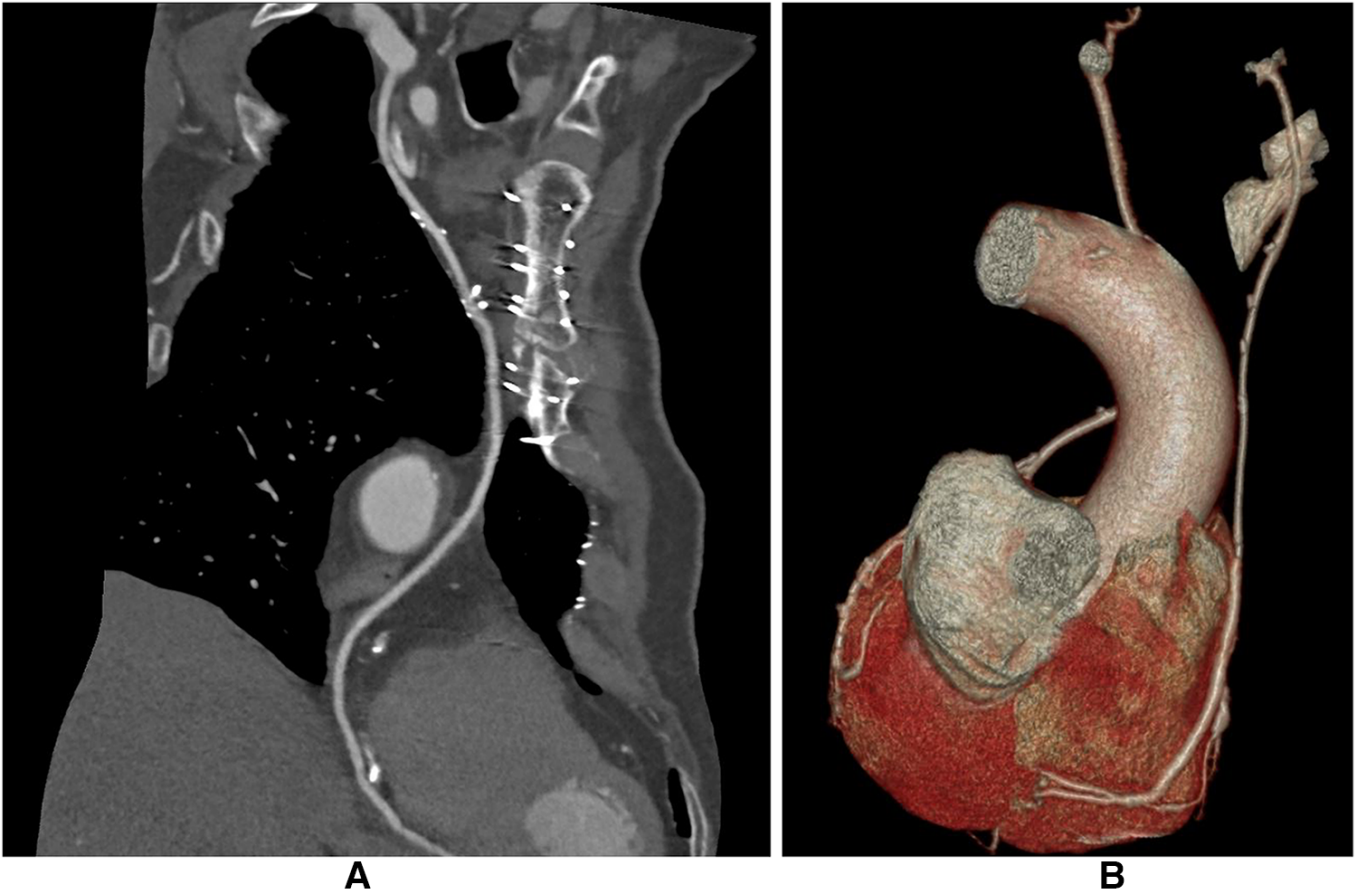
CCTA after I-graft RITA-SVG distally anastomosed to PDA. The diameter of the SVG has with time the trend to become equal to that of RITA. (**A**) After 123 months the size of the SVG is similar to that of RITA. (**B**) After 138 months the size of the SVG is still slightly larger than the RITA. CCTA, computed coronary tomographic angiography; RITA, right internal thoracic artery; SVG, saphenous vein graft; PDA, posterior descending artery.

Postoperative CCTA was performed in 132 patients, 64 in the I-graft group and 68 in the Ao-group (Figure 3). Patients in the I-graft group had a mean follow-up time significantly longer, 9 (7–11) vs. 6 (4–9) years, *p* < 0.0001. The 10-year Kaplan–Meier estimate of SVG patency was 70.4% ± 5.2, higher in the I-graft group than in that in the Ao-graft group (82.8% ± 6.5 vs. 58.8% ± 7.4, *p* = 0.0026).

The inner lumen diameter of the SVG measured in the CCTA was larger in the Ao-graft group than that in the I-graft group (3.4 ± 0.6 vs. 2.7 ± 0.4 mm, *p* < 0.0001). In contrast, the inner lumen diameter of the RITA in the I-graft group was 2.4 ± 0.3, similar to that of the LITA in the Ao-graft group, 2.5 ± 0.4 (*p* = 0.1180).

## Discussion

The SVG is the most commonly used graft for bypassing stenosed coronary arteries. Although there is growing evidence for more extensive use of ACs, the use of more than one arterial conduit (AC) (the LITA) is still limited. In addition, the results of ACs grafted into the RCAs are not perfect. In a recent meta-analysis, Yoshida et al. (11) showed that the 10-year estimates for patency of the RCAs in patients selected by propensity matching were similar in RA and SVG (78.2% vs. 79.5%, log-rank *p* = 0.600) when the ascending aorta was blood source. In a randomized trial in which the RCAs was grafted by the SVG, right gastroepiploic artery (RGEA), or RITA (as a Y-graft from the LITA), Glineur et al. (12) found that SVG had a statistically and significantly lower occlusion rate and higher proportion rate of functional grafts at 3-year angiographic follow-up than RGEA and RITA (7% vs. 13% vs. 22%, *p* = 0.05, and 86% vs. 65% vs. 68%, respectively, *p* = 0.004). In addition, the use of ACs is not always possible. The RA must be grafted on arteries with severe stenosis (≥90%) to limit the failure rate and RITA often cannot reach the RCAs due to its length. SVG then remains the most realistic solution.

However, the problems begin during harvesting, removal, and storage, as the endothelial cells (ECs) of the SVG can be mechanically damaged at all these stages. Even when the SVG is harvested using the no-touch technique, probably the least invasive harvesting technique, 20% of ECs are lost (13). The same study showed that, when the vein was mechanically distended with saline, the loss of ECs, including the 20% at baseline, was 29% at a distension pressure of 50 mmHg, 54% at 100 mmHg, 75% at 150 mmHg, and 91% at 300 mmHg (13). However, other studies have shown that SVG dilatation at a pressure of 100 mmHg, similar to arterial pressure, does not cause damage to the vein (14, 15). Another weak point is the storage solution. The graft is not sufficiently protected by normal saline (16), but better protection can be achieved if the vein is stored in blood containing heparin and papaverine.

There is a wide range of improvements in the handling and preparation of the SVG that can be made to limit the metabolic decompensation of the graft (17, 18). However, surgeons’ awareness seems to be limited. In a recent survey in the United Kingdom (19), only 44% of the surgeons believed that vein integrity could affect vein patency, 35% that vein integrity could affect the long-term clinical outcome (>1 year), and only 28% that vein integrity could affect the early clinical outcome (<1 year).

When the SVG is anastomosed proximally to the ascending aorta, it is suddenly exposed to a completely different environment. For example, arterial saturation is higher than in the original position, which in itself can stimulate intimal hyperplasia (20, 21). The most important change, however, is the exposure to new mechanical forces.

The pressure within the SVG is systemic and higher than usual, even if the SVG in its natural position can be exposed to a non-pulsatile pressure of ∼70 mmHg.

The circumferential stress or wall tension depends on the pressure inside the vessel, on its radius, and on its wall thickness. The main consequence of a chronic increase in wall tension due to a chronic increase in blood pressure is a progressive increase in wall thickness to bring the circumferential tension back into the normal range (wall tension is regulated by Laplace's law, which is directly proportional to pressure and radius and inversely proportional to wall thickness). This is a delayed manifestation of vascular adaptation as the vessel needs to accumulate mass (22). It has been shown that increased pressure and cyclic stretch lead to a loss of nitric oxide (NO) release and increased adhesion of immunocytes in the SVG (23).

Shear stress (SS) is a frictional force between the circulating blood and the vessel wall that is directly proportional to the velocity of the blood flow and inversely proportional to the cube of the radius. Small changes in the radius therefore have a significant effect on the SS. In humans, the SS in the straight artery is between 10 and 70 dynes/cm^2^. However, when the flow is disturbed, as in branches, bifurcations, and the inner curves of the artery, the net SS value is always less than 4 dynes/cm^2^ (24). In the venous system, the SS value is low, ranging between 1 and 6 dynes/cm^2^ (25), with an average of 2.2 dynes/cm^2^ in the SV (26).

Although far less than wall stress, SS is the mechanical force that drives the adaptation of vessel size to changes in flow. The ECs keep the SS stable (higher than 10 dynes/cm^2^) and respond to changes in blood flow by regulating the secretion of vasodilators, such as NO, and vasoconstrictors, such as endothelin-1. As the amount of blood flow is in the numerator and the cube of the radius in the denominator, the vessel enlarges when blood flow increases and vice versa. It is generally assumed that SS has a predominant influence on the vessel radius and circumferential wall stress on the wall thickness and stiffness.

The pattern of blood flow within the SVG is definitely different when it is anastomosed to the aorta than in its natural position. The blood flow in the arteries is unsteady as its velocity increases and decreases during systole and diastole. This results in an inconstant but unidirectional laminar and atheroprotective flow that promotes a quiescent state in ECs and suppresses proliferation and apoptosis compared to static or very low flow conditions (27, 28). Laminar flow with SS in a physiologic range maintains vascular homeostasis and is atheroprotective. On the contrary, dysfunction of ECs caused by non-laminar flow leads to atherogenesis and thrombosis (24).

To summarize, the SVG is subjected to higher pressure, pulsatile flow associated with a circumferential strain of 10%–15% (29) and to different SS magnitudes. The interaction of these three mechanical forces is the basis of the patency of the SVG.

When connected to the aorta, flow in the SV increases, but because of its large diameter, the increase in SS is modest and always lower than physiological arterial SS values, which range from 10 to 24 dynes/cm^2^, being low when <10 and high ≥25 dynes/cm^2^ (30). Isobe et al. (31) calculated that the SS values ranged between 2.1 ± 0.3 dynes/cm^2^ when SVG was grafted into a posterolateral branch and 3.6 ± 0.6 dynes/cm^2^ when grafted into the LAD. The same authors showed that SS in the LITA was 13.8 ± 1.1 dynes/cm^2^ when grafted into the LAD, almost four times higher than in SV grafted into the same coronary artery (31). The same results have been reported by others. Shimizu et al. (32) found that 1.5 years after surgery, SS in SVG was 5 ± 2 dynes/cm^2^, regardless of whether the degree of coronary stenosis was 50%–75% or >75%. Other groups reported slightly higher SS, generally below the threshold of 10 dynes/cm^2^ (26, 33, 34). Thus, the ECs of SVG proximally anastomosed to the ascending aorta are exposed to a higher SS than in the original territory, but still in the low range, which is generally considered atheroprone status. Low SS has many other disadvantages. It has been experimentally demonstrated (35) that the value of SS is inversely correlated with the extent of intimal hyperplasia and that arterial SS (∼9 dynes/cm^2^), but not physiologic venous SS (∼1 dynes/cm^2^) (36), can abolish intimal proliferation.

Another adverse effect (37, 38) of low SS is the downregulation of endothelial nitric oxide synthase (eNOS) expression (39–41), which plays a crucial role in maintaining coronary arterial vasodilation through the generation of NO. Loss of NO impairs the normal dilatory response of coronary arteries to endothelium-dependent vasodilators and causes vasospasm. In addition to its vasodilatory effects, NO has other important functions. Endothelium-derived NO inhibits platelet aggregation (42), neutrophil adhesion (43, 44), macrophage adhesion, and chemotaxis (45) and inhibits vascular smooth muscle proliferation (46, 47). It should be borne in mind that the amount of NO secreted by the ECs of the SVG is far less than that of the LITA (48–50), either at rest or upon stimulation (51). In addition, NO production was almost eliminated in conventionally harvested and stored SVG (52). The SVG is then, since the beginning, at risk for early thrombosis and has all the prerequisites to start the process that has, as a goal, intimal hyperplasia and late failure. In summary, the availability of NO is greatly reduced in the SVG after insertion from the aorta into the coronary circulation.

However, if the SVG is connected to an ITA, this process is attenuated or abolished. ITA is a drug-eluting graft and has active biological functions secreting bioactive molecules such as NO and other molecules including microRNAs with anti-proliferative and anti-atherosclerotic properties (53). The ITA can supplement the grafted territory with NO, as shown by Tarr et al. (54). These authors measured *in vivo* the NO level (nitrite) in the left anterior descending vein. The mean values of nitrite increased from 44.8 ± 4.9 to 70.7 ± 8.1 μMol (*p* < 0.001) before and after LITA to LAD anastomosis. The continuous secretion of NO by the ITA may be the basis for the known protection of the grafted coronary area from atherosclerosis, which is protected by the ITA or other arterial conduits such as RA (55). The SVG can then benefit from the same mechanism as the coronary artery to which the graft is anastomosed. The NO then can be delivered from the ITA to the SVG, as the latter is the pathway between the ITA and the target coronary bed, stabilizing the ECs and reducing or eliminating most of the consequences of the new mechanical environment. It has also been hypothesized that the compliance of the ITA, which is upstream of the SVG, may act in part as a shock absorber for the high pressure to which the SVG is subjected (53, 56). The continuous supply of NO allows the SVG to maintain its integrity while the adaptation process continues.

The patency of the SVG or of the RITA when grafted as a Y-graft to the LITA has been shown to be similar after 10 years (7). In addition, the diameter of the SVG decreases when it is connected to the LITA as a Y-graft, with a parallel increase in SS 1 year after surgery. In 23 patients intraoperatively and 6 patients at 1 year, the SVG diameter decreased from 3.2 ± 0.4 to 2.9 ± 0.4 mm, and SS increased from 4.3 ± 3.0 to 21.6 ± 16.3 dynes/cm^2^ (57). Intimal hyperplasia was not the cause of the decrease in diameter as the same group (58) performed a quantitative angiography and an intravascular ultrasound examination in 28 patients after surgery and 1 year postoperatively. The inner diameter of the SVG lumen decreased from 3.58 ± 0.61 to 2.71 ± 0.42 mm. Comparing the SVG and the proximal LITA, the intima-media thickness was 0.31 ± 0.12 and 0.23 ± 0.08 mm, respectively, and the ratio of intima-media thickness to vessel diameter was, in percentage, 15.91 ± 5.00 and 13.55 ± 5.08, respectively, with no statistical difference. Then the SVG, when used as a Y-graft from the LITA, reduces the inner lumen diameter without abnormal intima-media thickening, with the aim of increasing the SS to arterial values. This process may change the phenotype of the SVG from venous to arterial and may be the basis for the high long-term patency rate, demonstrated by long-term angiographic controls (7). Similar data were shown by Lobo Filho et al. (59) at 94 ± 49 months after surgery in 14 patients who underwent CABG using a composite LITA-SVG as Y-graft and an SVG from the aorta on the RCAs. The mean inner diameter of the SVG was 2.31 ± 0.55 mm with proximal anastomosis to the LITA and 3.39 ± 0.66 mm with proximal anastomosis to the aorta.

In our study, the SVG was connected end to end with the RITA stump for RCAs grafting, to increase the rate of anaortic myocardial revascularization on a beating heart. Our philosophy was to take advantage of the position of the RITA in the arterial system. The flow pattern in the ascending aorta changes after each bifurcation, and the oscillatory SS at this level attenuates the kinetic energy present in the ascending aorta. Then the RITA, being a third-order branch (ascending aorta, brachiocephalic trunk, right subclavian artery), has the advantage of being exposed to a lower mechanical load which, partially absorbed by its compliance, is transmitted to the SVG perhaps even more attenuated. NO secreted by the RITA is transmitted to the coronary bed via the SVG, which can also benefit from the NO supplementation to maintain quiescent its ECs. When this happens, the ECs of the SVG can adapt, if necessary, the diameter of the graft to the amount of blood flow inside the graft, balancing the secretion of NO and ET-1.

Although we did not actively measure the SS, it is clear that the SVG tends to adapt to RITA size and sometimes the two vessels are difficult to distinguish (Figure 3A), in a process similar to that described by Huang et al. (57, 58) and Lobo Philo et al. (59). This resulted not only in a higher 10-year patency compared to SVG from the aorta but also in a higher freedom from MACE, as the I-graft most likely behaves more like an arterial graft than a hybrid graft. These data are the result of a strategy that may improve the patency rate of SVG to RCAs.

Analysis of the two groups showed improved survival and freedom from MACE in the patients in whom the I-graft was used to graft the RCAs. The reason for these data may be the possibility that the I-graft ultimately behaves like an AC and provides the benefit of complete arterial revascularization. On the other hand, it cannot be excluded that propensity matching is not able to avoid all biases associated with patient selection, as only a randomized controlled trial can.

The idea of extending the RITA with the SVG is not new. It was used when the ascending aorta was heavily calcified or according to surgeon's preference. Benussi et al. (60) analyzed their patients with multivessel disease who underwent surgery with BITA grafting between 2012 and 2016 and compared patients in whom the RITA + SVG I-graft (*n* = 205) was used with all others (*n* = 685). Early and late outcomes were similar in both groups. Computed tomography was performed after a median of 4 years in 60 patients (6.7%). The patency rate of the I-graft was 88.3%.

Although the problem of SVG integrity is complex and cannot be simply linked to a single factor, we believe that the common denominator between the different techniques to improve SVG patency could be based on NO supplementation by the ITA at a time when the SVG is exposed to the disadvantages of a strong reduction in NO secretion by the activated ECs. A similar mechanism can be at the basis of the better patency of the SVG harvested with the no-touch technique. This aortocoronary graft may rely mainly on NO supplementation by the perivascular fat (49), making the NO supplementation hypothesis a unifying theory for the improvement of the future of the SVG. In favor of this hypothesis, fertile females are protected by atherosclerosis via activation of selective nitric oxide-releasing selective estrogen receptors in endothelial cells (61–63).

Other ACs were used to extend the RITA. The I-graft RITA + RA was mainly described in case reports, and the initial results were mostly described. The longest follow-up was published by Gatti et al. (64) who reported seven cases of RITA + RA with anastomosis to the RCAs, all of which were patent after a follow-up period of 1.2–5.7 years. Another AC, the RGEA, was used to prolong RITA. Shirakawa et al. (65) reported on 104 patients in whom the I-graft was anastomosed to different systems. Early patency was assessed in 54.8% of cases, with a patency rate of 100% for the LITA and 95.5% for the I-graft RITA + RGEA. It is noteworthy that the RA and RGEA are less flexible than the SVG, as their length is fixed, whereas the SVG can be harvested as needed. In addition, both ACs have an optimal flow when the coronary stenosis is 90% or more, a limitation that the SVG does not have.

This study has many limitations, as it is retrospective and observational. Propensity matching is generally not able to avoid all biases associated with patient selection. In particular, it is possible that some of the covariates that could have a stronger influence on the patency rate of conduits, such as female gender and diabetes, were not properly captured by propensity matching. Our results must be considered as a hypothesis and need confirmation in randomized clinical trials.

Graft patency was studied in a small number of patients, so any conclusions can only be speculative. In addition, the patency of the graft depends on many factors, such as coronary resistance, the extent of the grafted area, the severity of the coronary stenosis, technical factors, the angle of the anastomosis (the smaller, the better) (66, 67).

The strength of this study is the strategy of the RCAs grafting based on an arteriovenous conduit, which could reduce the failure rate of the SVG when anastomosed to the aorta. The hypothesis that shifting the blood source of SVG from the aorta to the ITA may lead to a different hemodynamic pattern and an anti-atherosclerotic profile is attractive but requires further data to be confirmed.

In summary, SVG may be an optimal graft for RCAs when connected to RITA. This strategy has important clinical implications. Not only can any aortic manipulation be avoided, reducing the rate of ischemic cerebral events, but there is also the possibility that this arteriovenous conduit can behave like an AC, because the SVG is subjected to low-grade mechanical stress and is continuously replenished by NO from the RITA. When connected to the LITA as a Y, it is possible that the SVG undergoes progressive arterialization, as the lumen decreases without intimal hyperplasia and the flow velocity and SS increase to arterial levels. We can assume that this process also occurs in the I-graft, as the mean inner lumen diameter is only slightly larger in the SVG than in the RITA. On the contrary, when the SVG is anastomosed proximally to the aorta, the reduction of the lumen is caused by the development of intimal hyperplasia, a process that gradually leads to graft failure.

Re-evaluating the role of SVG in coronary surgery means understanding the causes of improved patency rates associated with different strategies. The role of NO and the progressive increase in SS due to SVG arterialization may change our perspective on the use of conduits in myocardial revascularization. This must be the goal of further studies, to explore the physiology of this arteriovenous conduit, which may be the most effective graft for RCAs but also for other coronary territories.

## Data Availability

The raw data supporting the conclusions of this article will be made available by the authors, without undue reservation.
